# Analysis of Influencing Factors of CBOW Model in Natural Language Processing Based on Quantum Neural Network

**DOI:** 10.3390/e28060613

**Published:** 2026-05-29

**Authors:** Meng Zhang, Jian Kang, Bing Han, Qian Wu

**Affiliations:** Division of Quantum Information Technology Standardization, China National Institute of Standardization, Beijing 100191, China; kangjian@cnis.ac.cn (J.K.); hanb@cnis.ac.cn (B.H.); wuqian@cnis.ac.cn (Q.W.)

**Keywords:** quantum neural network, CBOW model, natural language processing, influencing factor, performance optimization, quantum circuit

## Abstract

To address the problems of the limited feature extraction capability and insufficient training efficiency of the traditional Continuous Bag-of-Words (CBOW) model in Natural Language Processing (NLP), the Quantum Neural Network-enhanced CBOW model (QNN-CBOW) integrates Quantum Neural Networks (QNN) with the CBOW model, effectively enhancing training performance. This work aims to systematically investigate the sensitivity and influence patterns of key factors (activation function type, number of quantum feature extraction layers, context window size, and quantum gate noise level) on model behavior under controlled small-scale simulation conditions. Comparative experiments are carried out using the control variable method to clarify the influence mechanism of each factor. This paper presents a NISQ-era proof-of-concept study, which provides a theoretical basis and practical reference for the fusion and optimization of quantum neural networks and traditional NLP models.

## 1. Introduction

As a core direction in the field of artificial intelligence, Natural Language Processing (NLP) [1,2,3,4,5,6] aims to enable computers to understand and generate human language. Word embedding, as the foundation of NLP tasks, directly determines the performance upper limit of subsequent tasks. As one of the core architectures of Word2Vec, the Continuous Bag-of-Words (CBOW) model predicts the central word through the linear combination of contextual words, offering the advantages of simple structure and high training efficiency. It is widely used in text classification, sentiment analysis, machine translation and other tasks. However, the traditional CBOW model relies on the fully connected layer of classical neural networks for feature extraction, which suffers from defects such as limited feature expression capability, and difficulty in capturing complex semantic correlations between words. The model tends to converge to local optima during training, limiting its global optimization capability, and its performance is limited when processing complex long texts or professional domain corpora.

As an emerging computing paradigm, quantum computing exhibits great potential to surpass classical computing in processing high-dimensional data and complex nonlinear problems by virtue of its unique physical properties such as quantum superposition and quantum entanglement. The Quantum Neural Network (QNN) combines the advantages of quantum computing with the structure of classical neural networks, and constructs feature extraction and mapping modules using quantum circuits, which can effectively improve the model’s ability to process high-dimensional data and reduce model complexity. In recent years, the application of quantum neural networks in the field of NLP has gradually emerged. Researchers have attempted to fuse quantum computing with word embedding models, Transformer models and other architectures to build quantum-enhanced NLP models, and have achieved certain research progress [7,8,9,10]. Proposed is a quantum natural language processing integrated geospatial query system built on Python and NLTK, which adopts toponym recognition, Flask UI and fuzzy location matching to optimize query processing, remedy the defects of traditional systems and provide users with an intuitive experience and context-rich results from multi-source data [11]. A novel approach leveraging quantum natural language processing, optimized pre-trained language models, quantum embedding and quantum feature extraction is proposed to enhance the accuracy, speed and depth of patient-clinical trial matching, bridge the gap between patients and medical research, and enable fast, precise key information mining from massive healthcare datasets [12]. A method is proposed to map classical NLP word and text embedding models such as fastText, GloVe, Numberbatch and BERT into quantum states via amplitude encoding, which achieves high vector compression while well-preserving semantic relational properties and information integrity, enabling quantum computation of semantic similarity by reusing existing classical embedding resources [13]. A study is proposed to enhance the noise robustness of Quantum Natural Language Processing models for sentiment classification using the Lambeq library, which evaluates SPSA, Nelder–Mead and Adam optimizers under diverse noise conditions and verifies that Adam achieves far higher accuracy and stability in both noiseless and noisy environments, providing valuable support for practical QNLP deployment on noisy quantum hardware [14]. A T5-based grammatical error correction system integrated with ERRANT error classification is proposed to boost correction performance and help users grasp grammatical errors, while exploring QNLP and quantum networking for secure, context-aware language processing beyond classical computing limits [15]. A quantum computing and NLP integrated framework is proposed for efficient cyber threat analysis, leveraging quantum algorithms and BERT-based models to achieve faster processing, high identification accuracy and lower false positives for next-generation cybersecurity [16]. A quantum-accelerated hyperparameter tuning framework is proposed to dynamically optimize NLP models via quantum neural networks, delivering faster tuning and stronger adaptability to streaming textual data [17]. A quantum-classical A-QLSTM-A model is proposed to address the limitations of biomedical LLMs, achieving outstanding accuracy for interpretable early lung cancer detection and clinical decision support [18]. Quantum word embedding models can realize richer semantic representation through quantum state superposition, and the parallel computing characteristics of quantum circuits can improve model training efficiency. However, these early works are conceptual and exploratory, without conclusive evidence of quantum advantage or practical applicability. Relevant research is still in the initial stage, and the influence mechanism of each key parameter on model performance has not been systematically analyzed, nor has the optimal configuration scheme of each factor been clarified.

The advancement of Noisy Intermediate-Scale Quantum (NISQ) devices has laid a preliminary hardware foundation for the implementation of quantum-enhanced natural language processing (NLP) models. Nevertheless, constrained by the limited number of qubits, inherent noise in quantum gate operations, as well as restrictions on circuit depth and width, a rational parameter configuration is pivotal to the effective deployment of such models on NISQ hardware. Synergistic interactions between quantum parameters—including quantum layer count (corresponding to circuit depth) and word embedding dimension (corresponding to circuit width)—and classical hyperparameters such as activation function selection and context window size exert intricate impacts on model performance, calling for systematic empirical investigation.

Against this backdrop, this paper presents a proof-of-concept study tailored for the NISQ era. Adopting the controlled variable method for comparative experiments, we systematically explore both the qualitative and quantitative effects of four core factors on the QNN-CBOW framework: activation function types, the number of quantum feature extraction layers, context window sizes, and quantum gate noise intensities. It should be clarified that all experiments in this work are conducted on classical quantum simulators with self-built miniature corpora. This study aims to empirically document and theoretically analyze the parameter dependence of hybrid quantum-classical CBOW variants under controlled experimental settings. We neither claim quantum advantage nor verify the generalization capability and performance improvement of the proposed algorithm in practical real-world NLP tasks.

## 2. Theoretical Foundation

The traditional CBOW model’s core idea is to predict the central word through the distributed representation of contextual words, and optimize the training process by adopting Negative Sampling or Hierarchical Softmax, which solves the problems of the curse of dimensionality and low training efficiency of traditional word embedding models. Compared with the Skip-gram model, the CBOW model is more suitable for large-scale corpora dominated by high-frequency words with a lower computational cost. It focuses more on capturing the overall context, but its performance is slightly weaker in capturing the semantic correlations of low-frequency words.

CBOW is a word embedding model that predicts the central word based on context. Its core idea is as follows: first, a text sequence is given. Then, with a certain central word as the prediction target, the probability distribution of the central word is predicted by using the context words within a certain range before and after the central word through linear combination and nonlinear mapping; and finally, the distributed representation (word vector) of the word is obtained through training. The core assumption of the CBOW model is that the semantics of the central word are jointly determined by its contextual words, and the semantic information of contextual words can be transmitted to the central word through linear combination.

The structure of the traditional CBOW model mainly includes three parts: the input layer, the hidden layer and the output layer, which are detailed as follows:

- Input layer: This layer takes the one-hot vectors of context words as input. Assuming the context window size is *k*, the input layer comprises 2*k* one-hot vectors. Each vector has a dimension equal to the size of the vocabulary. For instance, for the sentence “I love natural language processing”, when the window size is 2, the context words are “I, love, language, processing”, and the central word is “natural”. The input layer consists of the one-hot vectors of these four context words.
- Hidden layer: The 2*k* one-hot vectors in the input layer are mapped to low-dimensional word vectors through an embedding matrix. Then, these word vectors undergo average pooling to obtain the average word vector of the context words, which is used as the output of the hidden layer. The dimension of the hidden layer, which is one of the key parameters of the model, corresponds to the dimension of the word vectors.
- Output layer: The softmax function is employed to map the output of the hidden layer to the probability distribution of the central word. The dimension of the output layer equals the size of the vocabulary. By calculating the cross-entropy loss between the predicted probability and the true label, and updating the model parameters through backpropagation, we ultimately obtain the distributed representation of each word.

The training process of the traditional CBOW model mainly includes two stages: forward propagation and backpropagation.

- Forward propagation: The one-hot vectors of context words are input. They are mapped to low-dimensional word vectors through an embedding matrix. After average pooling, the context average word vector is obtained. The probability distribution of the central word is output through the softmax function.
- Backpropagation: The cross-entropy loss between the predicted probability and the true label is calculated. The embedding matrix and hidden layer parameters are updated using gradient descent to minimize the loss function until the model converges. To improve training efficiency, the traditional CBOW model often employs negative sampling or hierarchical softmax techniques to replace the traditional softmax function, thereby reducing computational complexity.

In recent years, researchers have proposed various improvement schemes to address the shortcomings of the traditional CBOW model. First, by introducing attention mechanisms, the model’s focus on key contextual words is enhanced. Second, by combining deep learning architectures such as Convolutional Neural Networks (CNN) and Recurrent Neural Networks (RNN), the model’s feature extraction capability is improved. Third, by incorporating domain knowledge, the semantic expression of word embeddings is optimized. However, these improvement schemes are all based on classical computational frameworks, making it difficult to break through the inherent limitations of classical neural networks in high-dimensional semantic space mapping.

Quantum Neural Network (QNN) takes qubits as the basic computing units, and it constructs quantum circuits through quantum gate operations to realize feature extraction and the mapping of data. Its core advantage is that it can process multiple high-dimensional features simultaneously by using quantum superposition states, and capture complex correlations between features by using quantum entanglement, which can effectively improve the model’s ability to process high-dimensional semantic data. In recent years, the research on the application of quantum neural networks in the field of NLP has gradually gained momentum, mainly focusing on three directions: the first direction is quantum word embedding. By representing words with quantum states, a high-dimensional semantic space is constructed to enhance the semantic expression ability of word embeddings. For example, the QCSE model achieves superior word embedding effects through quantum context-sensitive encoding. The second direction is quantum-enhanced text classification. Quantum neural networks are used as feature extraction modules, integrated with traditional classifiers, to improve classification accuracy. The third direction is quantum language models. Quantum circuits are utilized to construct language generation models, reducing model complexity.

A QNN is a new type of neural network that fuses quantum computing with classical neural networks. Its core idea is to use quantum characteristics such as qubits, quantum superposition and quantum entanglement to construct quantum feature extraction modules to replace or enhance the feature extraction capability of classical neural networks, while retaining the training and optimization framework of classical neural networks to achieve the efficient processing of high-dimensional data.

The structure of a QNN mainly includes three parts: the quantum input layer, the quantum feature extraction layer and the classical output layer, which are detailed as follows:

- Quantum input layer: Classical data, such as word vectors of text, are encoded into quantum states, achieving the mapping from classical data to quantum states. Common encoding methods include angle encoding, amplitude encoding, and so on.
- Quantum feature extraction layer: This layer consists of a quantum circuit composed of multiple quantum gates, serving as the core module of the QNN. Quantum state transformation is achieved through the operation of quantum gates, such as rotation gates and CNOT gates. It can extract quantum features of data to capture complex correlations between data. Factors such as the number of layers in the quantum feature extraction layer (corresponding to the depth of the quantum circuit) and the type and quantity of quantum gates directly determine the feature extraction capability and computational complexity of the QNN. The more quantum layers there are, the greater the depth of the quantum circuit, and the stronger the feature extraction capability. However, this also increases computational complexity and quantum noise accumulation.
- Classical output layer: This layer converts the quantum state measurement results output by the quantum feature extraction layer into classical data. Then, through the fully connected layer and the softmax function of the classical neural network, it achieves the prediction of the target task. At the same time, the prediction results are fed back to the quantum feature extraction layer to update the model parameters.

The training process of the QNN integrates the ideas of quantum computing and classical optimization, mainly including two stages: quantum forward propagation and classical backpropagation:

- Quantum forward propagation: Classical input data is encoded into quantum states. Quantum features are extracted through quantum gate operations in the quantum layer. Then, the quantum state is measured to obtain classical feature data. This data is input into the classical output layer, and the prediction result is obtained.
- Classical backpropagation: The loss function between the predicted results and the true labels is calculated. Using classical optimization algorithms (such as gradient descent), the gradient of the loss function with respect to the quantum layer parameters (such as the rotation angle of quantum gates) is computed. Then, the parameters of the quantum layer and the classical output layer are updated to minimize the loss function until the model converges.

Due to the randomness of quantum state measurement, it is necessary to measure quantum states multiple times in the training process of the QNN, and to take the average value as the output of quantum features. At the same time, the parameter update of the quantum layer is constrained by the quantum gate operation, and the value range and update step size of parameters need to be set reasonably to avoid quantum state distortion and noise accumulation. On NISQ devices, the parameter update of the quantum layer also needs to consider the limitations of the hardware, such as the number of qubits and the error rate of quantum gates. Gradient estimation methods such as SPSA is usually adopted to reduce the query overhead of quantum measurement and improve training efficiency.

The overall architecture of the QNN-CBOW model integrates the advantages of quantum neural networks and traditional CBOW models, mainly including five parts: the input layer, the quantum encoding layer, the quantum feature extraction layer, the classical semantic mapping layer and the output layer. The functions and roles of each layer are as follows:

- Input layer: It is consistent with the input layer of the traditional CBOW model. It takes the one-hot vectors of context words as input. The dimension of the input layer equals the size of the vocabulary. The number of inputs equals double the size of the context window.
- Quantum encoding layer: It maps the one-hot vectors of the input layer to a low-dimensional classical word vector through an embedding matrix. Then, it employs an angle encoding method to encode the classical word vector into a quantum state.
- Quantum feature extraction layer: It consists of multiple quantum circuit modules. Each quantum circuit module includes single-qubit gates (rotation gates such as RX, RY, RZ) and two-qubit gates (such as CNOT gates). This layer is the core module of the QNN-CBOW model. Through the operation of quantum gates, it realizes the transformation of quantum states, extracts the quantum semantic features of words, and captures the complex associations between context words and the central word. The single-qubit gate is used to adjust the rotation angle of the quantum state, achieving fine-tuning of quantum features. The two-qubit gates are used to introduce entanglement between qubits, enhancing the ability to capture semantic associations between context words and the central word. The number of layers in the quantum feature extraction layer can be flexibly adjusted and is one of the influencing factors analyzed in this paper.
- Classical semantic mapping layer: It converts the quantum state output by the quantum feature extraction layer into classical data (quantum feature vectors). Then, through the fully connected layer, the quantum feature vector is mapped to a classical semantic vector with the same output dimension as the hidden layer of the traditional CBOW model, achieving the mapping from quantum features to classical semantic features. The role of this layer is to bridge the quantum layer and the output layer. It converts quantum features into classical data that can be processed by the classical output layer, while retaining the semantic information of quantum features and improving the predictive performance of the model.
- Output layer: It is consistent with the output layer of the traditional CBOW model. It employs the softmax function to map the classical semantic vectors output by the classical semantic mapping layer to the probability distribution of the target word (or the class probability distribution for text classification). The dimension of the output layer equals the size of the vocabulary (or the number of classes for text classification). By calculating the cross-entropy loss between the predicted probability and the true label, bac propagation updates all parameters of the model (including the embedding matrix, quantum layer parameters, and classical semantic mapping layer parameters) until the model converges.

**Figure 1:**
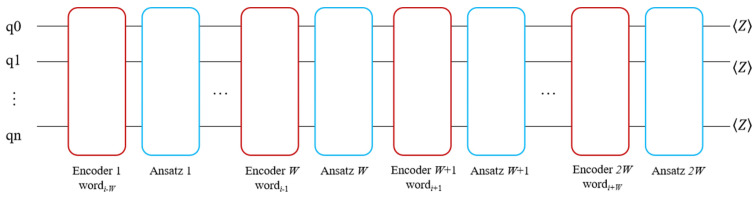
Schematic diagram of quantum circuit.

In this experiment, the quantum circuit shown in Figure 1 is used to build a quantum neural network to complete the word embedding task. Assuming the text window size is *W*, for the *i*th word, 2*W* quantum circuit groups (Encoder + Ansatz) are required.

## 3. Experimental Results

Based on the MindSpore Quantum programming toolkit [19], this paper conducts experimental research on the key factors affecting the CBOW model based on quantum neural network in NLP from the dimensions of activation function type, number of quantum feature extraction layers, context window size, and quantum computing hardware noise level, providing a reference for relevant research. Considering that the current quantum computing processor hardware is not mature enough to run complex algorithms, the experiments in this paper are conducted on a classical simulator for quantum circuits. The configuration of the classical simulator for quantum circuits is a 4-core CPU, 8 G memory and 256 G disk. Due to hardware constraints in the classical simulator for quantum circuits, we opted for custom English text rather than standard public NLP datasets. The corpus consists of several segments of professional text. After tokenization, it contains approximately 64 words, with a unique vocabulary size of 43. Given the simplicity of the corpus, we omitted complex preprocessing steps like stop-word filtering or semantic noise reduction. Instead, the focus was on validating the feasibility and convergence performance of the quantum CBOW model within a hybrid quantum-classical framework for word embedding tasks. Consequently, we conducted baseline comparisons with standard models such as the classic CBOW model.

To comprehensively evaluate the performance of the proposed Quantum Natural Language Processing (QNLP) model, we employ three core metrics to quantify performance across the dimensions of prediction error, classification accuracy, language modeling capability, and representation quality.

1. Loss Function

First, we utilize the Cross-Entropy Loss to quantify the divergence between the predicted distribution and the ground truth labels. In the computational process, the output layer initially generates raw, unnormalized scores, known as Logits. These Logits are subsequently transformed into a probability distribution *P* via the Softmax function. The loss is calculated as the negative log-likelihood of the true class, formulated as: $L=-log\left(P_{true}+\epsilon \right)$, where *ϵ* denotes a smoothing term introduced to prevent numerical overflow.

2. Accuracy

Second, Accuracy serves as the baseline metric for the classification task, directly reflecting the model’s discriminative power. This metric is derived from the Logits: the category corresponding to the maximum Logit value is selected as the prediction. A prediction is considered correct if and only if it matches the ground truth label *y*_true_. The calculation is defined as: $Accuracy=\{\begin{array}{cc} 1.0 & \mathrm{if}\;argmax(\mathrm{Logits})=y_{true}\\ 0.0 & \mathrm{otherwise} \end{array}$.

3. Perplexity (PPL)

Third, Perplexity (PPL), rooted in information theory, is employed to evaluate the uncertainty of the language model regarding the test data. It represents the exponential form of the cross-entropy loss, expressed as: $PPL=e^{Loss}$. A lower perplexity indicates that the model’s predicted distribution approximates the true distribution more closely, thereby demonstrating superior generalization capabilities.

**Figure 2:**
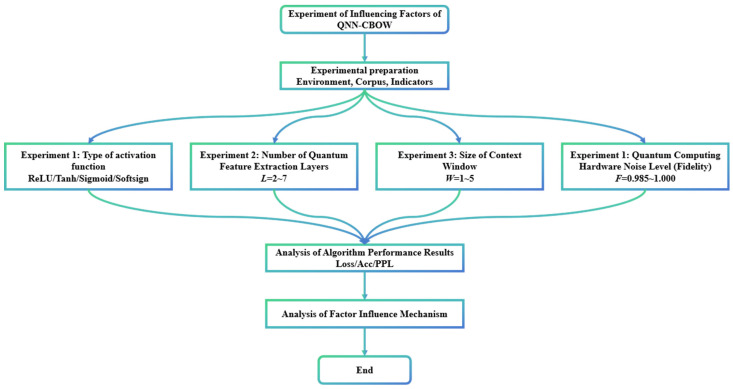
Schematic diagram of experimental process.

The experimental process is shown in Figure 2.

Due to limited computing resources in this experiment, large-scale computations on complex NLP datasets were not feasible. This study exclusively adopts a designated English text corpus as the sole data source to conduct comparative experiments between Quantum Neural Networks (QNN) and classical neural networks for natural language processing word embedding tasks. All experimental data were derived purely from the specified corpus, with no external public datasets or additional auxiliary data incorporated. The original text corpus is presented as follows: “We are about to study the idea of a computational process. Computational processes are abstract beings that inhabit computers. As they evolve, processes manipulate other abstract things called data. The evolution of a process is directed by a pattern of rules called a program. People create programs to direct processes. In effect, we conjure the spirits of the computer with our spells.”

Dataset construction, data preprocessing and sample extraction were implemented in full compliance with standard specifications. The detailed procedures are outlined below. First, the raw English corpus was preprocessed: the entire text was split into individual words via whitespace segmentation. Duplicate words were eliminated, and the remaining unique vocabulary was sorted in ascending order. A word-to-index mapping dictionary was then established to assign each distinct word a unique integer index. The finalized dictionary contains 61 unique words, corresponding to a dictionary size of 61.

For sample extraction, the sliding window sampling strategy of the Continuous Bag-of-Words (CBOW) model was adopted, with the sliding window size defined as *W*. Each sample consists of a target center word along with W context words on both its left and right sides. Sampling commenced from the *W*-th position and terminated at the penultimate *W*-th position in the segmented word sequence, generating paired samples of context word sets and center words sequentially. Each context set comprises 2*W* words evenly distributed on both sides of the center word. Ultimately, 58 valid samples were extracted from the original corpus to form the complete experimental sample set.

Targeted secondary preprocessing was performed on extracted samples to meet the input requirements of both quantum and classical neural networks. For quantum neural networks, the required number of qubits was calculated using the formula: $n_{qubits}=\left\lceil {log}_{2}\left(1+max(dictionary\;size)\right)\right\rceil$. Given the dictionary size of 61, the calculated qubit number was 6. Each integer index corresponding to context words was converted into a 6-bit binary string, with trailing zeros supplemented to reach the fixed length. Every binary digit (0 or 1) was further mapped to a corresponding quantum rotation angle (0 for digit 0, π for digit 1), forming a 6-dimensional angle encoding vector for each context word. Encoding vectors of all 2*W* context words were concatenated to form the network input vector, while the integer index of the center word served as the sample label, forming the training dataset for QNNs.

For classical neural networks, integer indices of context words were directly used as network inputs; the indices of all 2*W* context words constituted the input vector, and the center word index was taken as the label to build the training data for classical neural networks.

In terms of dataset partitioning, all 58 acquired samples were utilized entirely as the training set without being split into training, validation and test subsets. The rationale is as follows: the core objective of this experiment is to verify the practicability, training stability and convergence characteristics of QNNs in word embedding tasks, with the primary focus on comparing the training dynamics and convergence performance between quantum CBOW and classical CBOW models, rather than evaluating model generalization ability, robustness and practical application effectiveness. Therefore, validation sets for parameter tuning and test sets for performance assessment were unnecessary. Full-sample training maximizes the utilization of limited samples and guarantees sufficient model training as well as reliable convergence results.

All experimental hyperparameters and training configurations were standardized uniformly to ensure full experimental reproducibility. A set of baseline experiments was conducted first, followed by configuration adjustments to compare algorithm performance under varied parameter settings. The detailed baseline configurations are specified below.

Fixed global hyperparameters include: sliding window size = 2, word embedding dimension = 10, hidden layer dimension for both classical and quantum models = 128, quantum Ansatz circuit depth = 3, parallel threads for quantum simulators = 4, batch size = 3, and total training epochs = 350. Fixed random seeds were applied to standardize random processes including model initialization and data loading order, ensuring identical initial experimental conditions across all trials.

During training, the sparse Softmax cross-entropy loss function was uniformly adopted to quantify the discrepancy between model predictions and ground-truth labels. The momentum optimizer was selected with an initial learning rate of 0.01 and a momentum coefficient of 0.9. No additional optimization strategies such as learning rate decay or weight decay were deployed; model parameters were updated iteratively solely via fixed learning rate and momentum settings.

Detailed model training configurations are as follows: the quantum CBOW model ran in dynamic graph mode under the MindSpore framework, while the classical CBOW model operated in static graph mode, with all training procedures executed on CPU devices. Quantum circuit computations were realized via the mqvector quantum simulator with 4 fixed parallel threads to accelerate quantum simulation efficiency.

The Quantum Embedding (QEmbedding) layer was configured as follows: the Hadamard gate was used to initialize quantum states into uniform superposition states to enhance the expressive capability of QNNs. Afterwards, cascaded structures consisting of 2*W* groups of Encoder circuits and Ansatz circuits were integrated. Encoder circuits composed of RX rotation gates were set as non-trainable modules, solely responsible for quantum encoding of lexical information without parameter optimization. Ansatz circuits were constructed with RY rotation gates and CNOT gates in a layered structure, containing 6 RY gates per layer and adjacent-qubit CNOT connections arranged according to layer parity, with a total of 3 trainable layers. In the measurement phase, Hamiltonians matching the embedding dimension were constructed to measure the expected values of final quantum states under the Z-basis, which were then output as word embedding vectors.

The standardized optimization workflow is described as follows. First, initialize model parameters and optimizers, load preprocessed training data and construct dataloaders with shuffle disabled to maintain consistent data loading sequences in each training round. Enter the formal training loop, load batched training data iteratively and feed them into the model to obtain prediction outputs. Compute loss values between predictions and true labels, and update all trainable model parameters including quantum Ansatz circuit parameters and weight parameters of fully connected layers in classical networks via backpropagation with the momentum optimizer, while keeping Encoder circuit parameters frozen.

Real-time training monitoring was enabled via callback functions; training steps, elapsed time and real-time loss values were printed every 500 steps, and full loss records were archived for subsequent data analysis. Upon training completion, the total training duration and trainable parameter values of quantum circuits were exported, and loss convergence curves were plotted to finalize model training and experimental result documentation.

### 3.1. Type of Activation Function

This section analyzes the impact of different classical activation functions on the performance of the QNN-CBOW model. In the QNN-CBOW model, the activation function acts on the output of the fully connected layer of the classical semantic mapping layer, and its core role is to perform nonlinear transformation on the classical feature vectors obtained by quantum measurement, screen effective quantum semantic features and suppress noise. Due to the inherent randomness in quantum state measurement, quantum eigenvectors contain a certain amount of noise. Therefore, the nonlinear expression capability, gradient retention ability, and anti-saturation characteristics of the activation function directly determine whether quantum features can be effectively transmitted to the output layer, and whether gradients will disappear or explode during the model’s backpropagation process.

Four classical deep learning activation functions were selected in this paper, which are detailed as follows:

1. ReLU: A non-saturated, hard activation function with *f*(*x*) = max(0,*x*). It has high computational efficiency and can alleviate the gradient vanishing problem, which is the mainstream activation function of classical neural networks.
2. Tanh: A saturated soft activation function with $f(x)=\tan h(x)=\frac{e^{x}-e^{-x}}{e^{x}+e^{-x}}.$ Its output is normalized to [−1,1], with strong nonlinear expression ability. But it is prone to gradient saturation in regions with large absolute values.
3. Sigmoid: A saturated soft activation function with $f(x)\;=\frac{1}{1+e^{-x}}.$ Its output is normalized to [0,1] with a clear physical meaning (probability mapping), but the gradient saturation problem is more serious, which easily leads to gradient vanishing.
4. Softsign: A soft saturation activation function with $f(x)\;=\frac{x}{1+\left|x\right|}.$ Its output is normalized to [−1,1]. Compared with Tanh, its saturation speed is slower, the gradient vanishing is gentler, and it has a certain robustness to noise.

In the experiment, the number of quantum feature extraction layers is fixed at 2, the word vector dimension is fixed at 10, and the context window size is fixed at 2.

**Figure 3:**
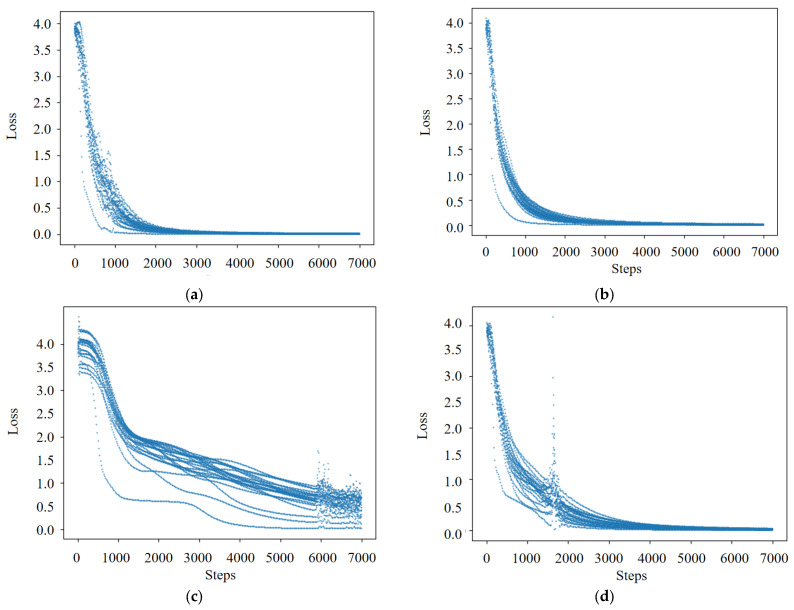
Variation in the loss value during the optimization process under different activation functions: (**a**) ReLU; (**b**)Tanh; (**c**) Sigmoid; (**d**) Softsig.

Figure 3 shows the variation in the loss value during the optimization process under the condition of selecting four activation functions: ReLU (a), Tanh (b), Sigmoid (c) and Softsign (d). In this experiment, the number of quantum feature extraction layers is fixed at 2, the context window is fixed at 2, and the word vector dimension is fixed at 10. It can be seen that the loss can converge rapidly when ReLU and Tanh are used as activation functions, and the loss value tends to 0 at Step = 3000. When Softsign is used as the activation function, the loss value fluctuates in the early stage of the optimization process but eventually converges, and the loss value tends to 0 at Step = 5000. In this experiment, the worst performance is exhibited by Sigmoid, whose loss value oscillates continuously during the optimization process and does not converge at Step = 7000. This is because the gradient of the quantum feature extraction layer itself possesses randomness and suffers from vanishing. After the saturation transformation of Sigmoid, the gradient can hardly be transmitted to the quantum feature extraction layer, resulting in the failure of effectively updating of the parameters of the quantum feature extraction layer. The model can only rely on the parameter fitting of the classical semantic mapping layer, thereby greatly reducing the optimization efficiency.

**Figure 4:**
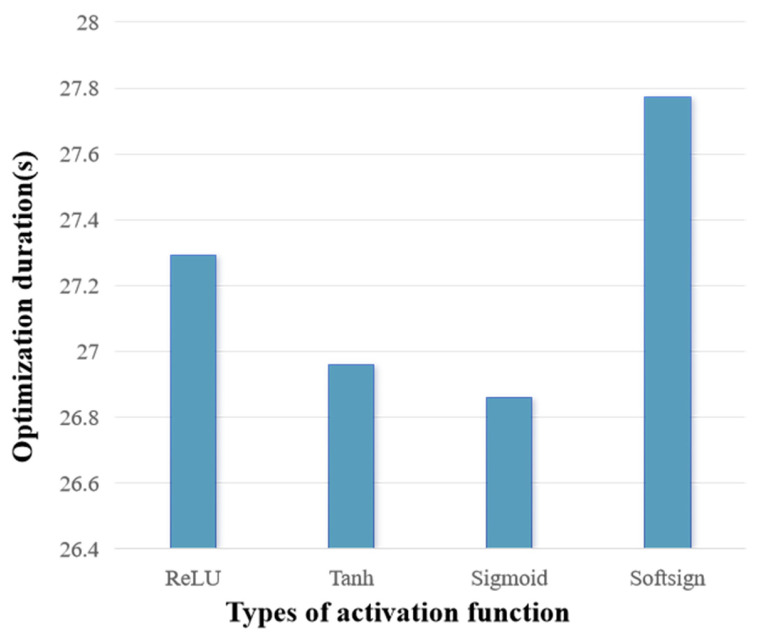
Impact of activation function types on the optimization duration.

Figure 4 shows the impact of activation function types on the optimization duration. It can be seen that for the same number of steps, the type of activation function has little effect on the overall optimization duration. This is because in the optimization process of the QNN-based CBOW algorithm, most of the time is consumed in the generation and simulation of quantum circuits, whereas the impact of the classical algorithm part is small. This can be clearly seen in the subsequent experiments on the number of quantum feature extraction layers.

**Figure 5:**
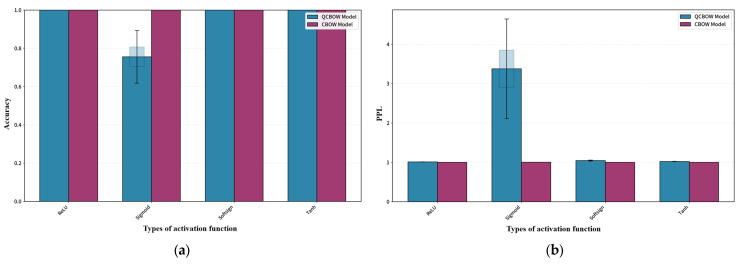
Impact of activation function types on the accuracy and PPL: (**a**) Accuracy; (**b**) PPL.

Figure 5 illustrates the impact of different activation functions on accuracy (a) and perplexity (PPL) (b). Multiple experimental trials were conducted to verify the stability of the convergence results. The plot displays the mean values, standard deviations (black error bars), and 95% confidence intervals (semi-transparent error bars), with results from the classical CBOW algorithm included for comparison.

Combined with the results in Figure 3, the loss of the QNN-CBOW model fails to converge when the Sigmoid function is adopted as the activation function. Consequently, its accuracy is noticeably lower than that achieved with other activation functions. The PPL results also reveal that the PPL value of the Sigmoid-based experiment is significantly higher than those of experiments using alternative activation functions. A PPL value of 1 represents the theoretical upper bound, indicating the model achieves 100% certainty and accuracy for every prediction, which is practically unattainable in real-world experiments. A PPL value between 1 and the vocabulary size signifies that the model has effectively learned grammatical rules and semantic representations. The closer the PPL value is to 1, the better the model’s overall performance. For instance, a PPL of 2 implies the model makes binary predictions with the same randomness as a coin flip, rather than guessing blindly among dozens of candidate words.

Additionally, it can be observed from Figure 3 that the classical CBOW algorithm maintains outstanding performance regardless of the activation function selected. In contrast, the QNN-CBOW model exhibits a far stronger dependence on the choice of activation function.

The nonlinear characteristics of activation functions are intricately coupled with the gradient propagation mechanisms of quantum neural networks. Their saturation properties and gradient magnitudes directly govern the effective transmission of quantum feature information, emerging as a critical factor determining the convergence performance of the QNN-CBOW model. Quantum neural networks leverage quantum superposition and entanglement to extract high-dimensional semantic features. When quantum features are mapped to the classical domain, they exhibit inherently small gradient magnitudes and a high susceptibility to gradient decay, imposing stringent requirements on the gradient preservation capabilities of activation functions. The Sigmoid function presents notable limitations in quantum settings due to its highly compressed output range and strong saturation behavior. Its pronounced saturation readily leads to near-vanishing gradients, impeding effective updates to quantum layer parameters during backpropagation. Consequently, the model relies solely on limited fitting by the classical semantic mapping layer, ultimately resulting in oscillating loss values, convergence difficulties, and a substantial decline in prediction accuracy. In contrast, ReLU, Tanh, and Softsign possess non-saturating or weakly saturating properties with superior gradient retention, effectively mitigating gradient decay, ensuring normal iteration of quantum layer parameters, and enabling stable convergence and high-performance outputs. The conventional CBOW model, by contrast, adopts a purely classical linear architecture where word embedding learning relies on the linear aggregation of contextual features. Nonlinear activation functions only act on auxiliary output layers, exerting minimal impact on core semantic representation. Thus, the model exhibits low sensitivity to activation function types, maintaining consistent performance across different activation choices.

### 3.2. Number of Quantum Feature Extraction Layers

The quantum feature extraction layers enhance the model expression capability by introducing quantum entanglement and parameterized circuits, achieving layer-by-layer refined extraction of language features from character level to context level. They fully leverages the advantages of quantum superposition, significantly improving the word embedding quality and semantic capture ability of the QNN-CBOW. On the basis of selecting ReLU as the activation function, this paper experimentally studies the impact of the number of quantum feature extraction layers (2 to 7 layers) on the algorithm performance. In the experiment, the word vector dimension is fixed at 10, and the context window size is fixed at 2.

**Figure 6:**
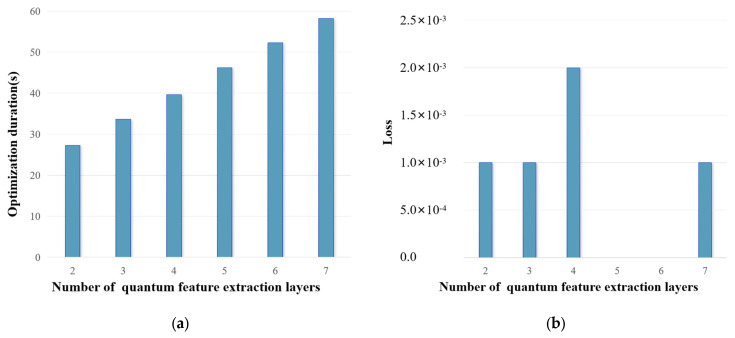
Impact of the number of quantum feature extraction layers on the optimization duration and loss value. (**a**) Optimization duration; (**b**) Loss.

Figure 6 shows the impact of the number of quantum feature extraction layers on the optimization duration and loss value (the loss value in the figure is the loss value at Step = 7000). It can be seen that with the increase in the number of quantum feature extraction layers, the optimization duration shows a linear growth trend. This is because the increase in quantum feature extraction layers leads to a linear growth of the quantum circuit depth, which also makes the running time of the quantum circuit increase linearly each time. When the number of quantum feature extraction layers is between 2 to 7 layers, a low loss value is obtained in all cases. Specifically, the loss value is very small when the number of layers is 4 and 5, indicating that the optimization result is very good.

**Figure 7:**
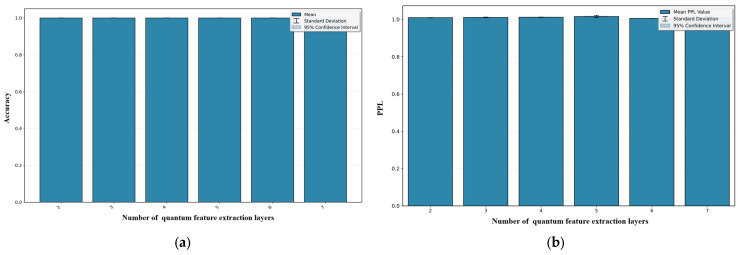
Impact of the number of quantum feature extraction layers on the accuracy and PPL: (**a**) Accuracy; (**b**) PPL.

Figure 7 depicts the impact of the number of quantum feature extraction layers on model accuracy (a) and perplexity (PPL) (b). Consistent with the results in Figure 6b, the number of quantum feature extraction layers exerts barely any influence on the predictive performance of the QNN-CBOW model. The model achieves a 100% accuracy rate across all repeated experiments. Although the PPL value fluctuates slightly, it remains close to 1 overall.

When using the QNN-CBOW algorithm, the number of quantum feature extraction layers must be selected appropriately. Increasing the number of quantum feature extraction layers within a certain range can improve the optimization effect, albeit at the cost of increased algorithm running time.

### 3.3. Size of Context Window

The context window size refers to the number of contextual words selected before and after the target word, which is a key hyperparameter of QNN-CBOW and directly affects the input dimension, training complexity and final word vector quality of the model. The context window size has a great impact on the output results. Too small a window results in insufficient context information, leaving the model unable to fully capture the semantic associations between words, which leads to a low discriminability of word vectors. Too large a window introduces irrelevant words from distant positions, increases noise interference, blurs semantic boundaries, and reduces the accuracy of the word vectors.

On the basis of selecting ReLU as the activation function, this paper experimentally studies the impact of the context window size ( from 1 to 5) on the algorithm performance. In the experiment, the word vector dimension is fixed at 10, and the number of quantum feature extraction layers is fixed at 3.

**Figure 8:**
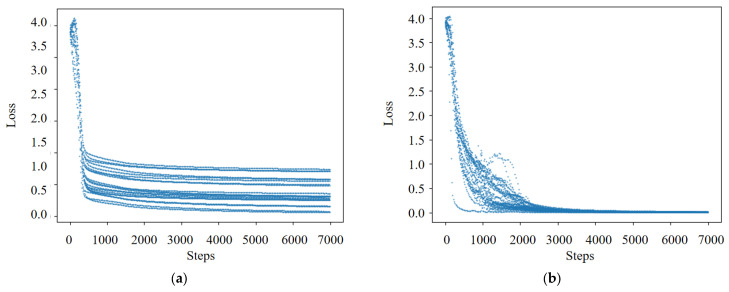

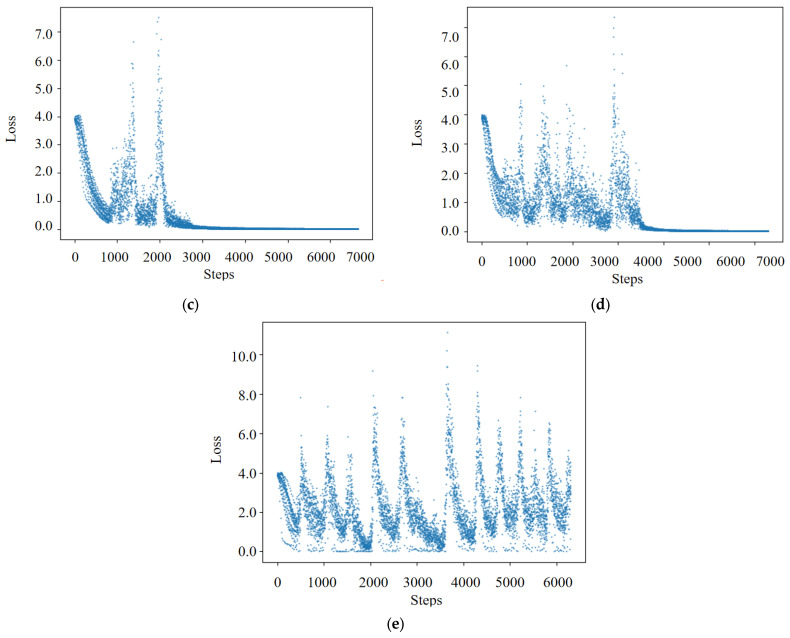
Variation in the loss value during the optimization process under different context window size. (**a**) Window = 1; (**b**)Window = 2; (**c**) Window = 3; (**d**) Window = 4; (**e**) Window = 5.

Figure 8 shows the variation in the loss value during the optimization process when the context window is set to 1 (a), 2 (b), 3 (c), 4 (d) and 5 (e). It can be seen that the convergence effect is the best when the window size is set to 2. When the window size is 1, due to insufficient contextual information, the training results frequently switch between multiple possible solutions. From the graph, it can be seen that the loss value curve splits into several parallel lines and never converges. When the window size is too large, the loss curve oscillates sharply multiple times during the training process due to the introduction of too much redundant semantic information. Especially when the window size is 5, the loss curve is always in an oscillating state and cannot converge.

**Figure 9:**
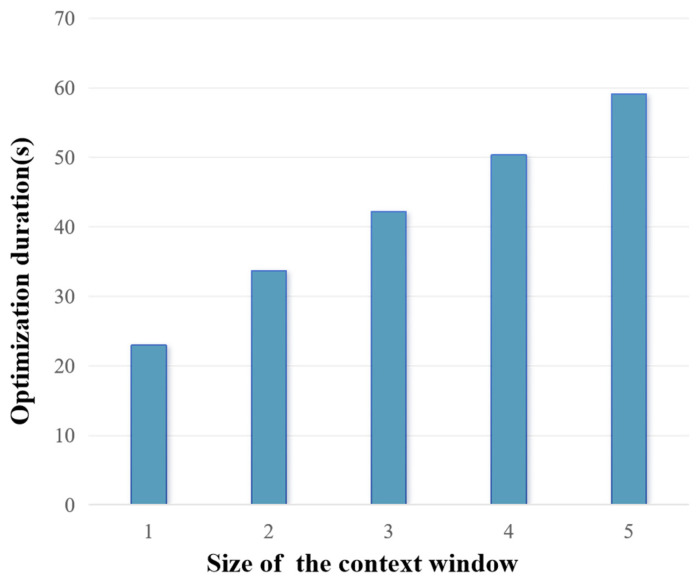
Impact of the context window size on the optimization duration.

Figure 9 shows the impact of the context window size on the optimization duration. It can be seen that the larger the context window size, the longer the time required for optimization. This is because the increase in the context window size leads to an increase in the number of input words, which in turn increases the number of parameters in the quantum circuit, and ultimately increases the running time of the quantum circuit.

**Figure 10:**
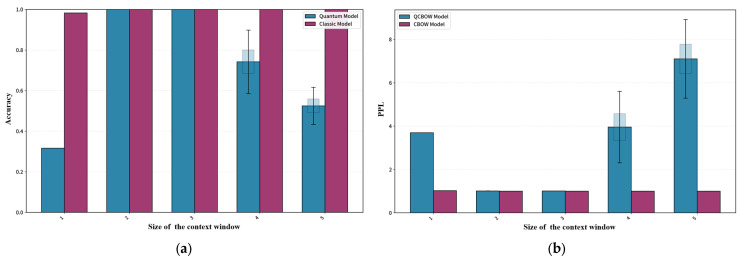
Impact of the context window size on the accuracy and PPL: (**a**) Accuracy; (**b**) PPL.

Figure 10 illustrates the effect of context window size on accuracy and perplexity (PPL). Consistent with the conclusions drawn from Figure 8, either an excessively small context window size (*W* = 1) or an overly large one (*W* = 4/5) impairs the predictive performance of the QNN-CBOW algorithm. In sharp contrast, the conventional CBOW model maintains consistently optimal performance across the window size range of *W* = 2 to 5, demonstrating remarkable robustness to variations in context window settings.

The underlying mechanism lies in the inherent structural difference between the classical CBOW and the quantum-enhanced QNN-CBOW. Classical CBOW relies on static linear embedding aggregation and has a strong generalization ability for local semantic windows; it can stably capture contextual semantics even with narrow or moderately expanded window sizes. By contrast, QNN-CBOW depends on quantum state superposition and entanglement to encode contextual information. When the window size is too small, the model lacks sufficient contextual semantic features for quantum state encoding; when the window size is excessively large, redundant noisy information is introduced into the quantum feature space, leading to quantum state aliasing and interference. This further degrades embedding quality and raises perplexity, thereby weakening the prediction accuracy and model stability.

### 3.4. Quantum Computing Hardware Noise Level

To further investigate how quantum computing hardware noise levels affect the performance of the QNN-CBOW algorithm, different levels of noise were introduced into a classical simulator for quantum circuits. This setup causes the fidelity of quantum logic gates to deviate from the ideal value of *F* = 1.

In this experiment, the word vector dimension was fixed at 10, the context window size at 2, the activation function as ReLU, and the number of quantum feature extraction layers at 3. This parameter combination delivered optimal performance under noise-free simulation conditions.

**Figure 11:**
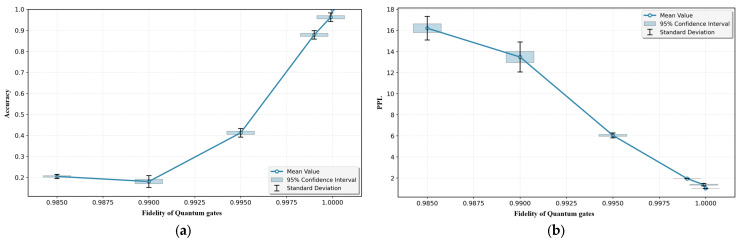
Impact of quantum computing hardware noise level on the accuracy and PPL: (**a**) Accuracy; (**b**) PPL.

Figure 11 presents the impact of quantum computing hardware noise levels on accuracy and perplexity (PPL). The experimental results indicate that the fidelity of quantum logic gates exerts a dramatic influence on the algorithm’s overall performance. When the fidelity is set at *F* = 0.9999, the average accuracy remains above 95%, with an average PPL of approximately 1.389. At a reduced fidelity of *F* = 0.995, the average accuracy drops below 50%, while the average PPL rises to over 5. As the fidelity further declines to *F* = 0.985, the PPL value approaches 20, nearly half the vocabulary size. At this noise level, the QNN-CBOW algorithm essentially makes random guesses over the entire vocabulary.

Quantum computing hardware noise primarily manifests as decoherence and gate operation errors, which directly distort the superposition and entanglement states constructed in the QNN-CBOW framework. High gate fidelity *F* = 0.9999 ensures quantum state evolution follows the theoretical design trajectory with minor interference only. The quantum feature encoding can effectively capture contextual semantic information, so the model maintains high prediction accuracy and low perplexity.

It should be pointed out that quantum bit gate errors accumulate gradually in the gate circuit. After performing *M* gate operations, its fidelity is only *F^M^*. As hardware noise increases and fidelity decreases, accumulated gate errors gradually corrupt quantum embedded representations. Quantum state aliasing and information distortion become severe, breaking the correlation between contextual features and target words. Once fidelity drops to *F* = 0.985, the effective semantic information carried by quantum states is almost completely submerged by hardware noise. The model loses the ability to extract valid context dependencies, resulting in its prediction performance degenerating into blind random guessing within the vocabulary space.

## 4. Conclusions and Future Work

This paper constructs a Quantum Neural Network-enhanced CBOW model (QNN-CBOW), systematically analyzes the impacts of key factors such as the type of activation function, the number of quantum feature extraction layers and the size of the context window on the model performance by using the control variable method, and clarifies the influence law and internal action mechanism of each factor.

The type of activation function has a significant impact on the convergence speed and accuracy of the QNN-CBOW model. The ReLU activation function is the optimal adaptive activation function; its non-saturated characteristics can effectively ensure the gradient transmission of the quantum layer, and suppress quantum measurement noise, resulting in the fastest convergence speed and the highest accuracy. The type of activation function only affects the gradient update stage of classical backpropagation, and has no significant impact on the model training time.

The number of quantum feature extraction layers has a nonlinear impact on the performance of the model. Appropriately increasing the number of layers within a certain range improves the quantum feature extraction ability, but at the same time, it leads to an increase in computational complexity. After the number of quantum feature extraction layers exceeds a certain range, the feature extraction ability does not significantly improve, and may even decrease. At this point, the complexity of calculations severely restricts the effectiveness of applications. In practical applications, various factors must be considered comprehensively.

The context window size is strongly correlated with the characteristics of the dataset. For the dataset selected in the experiments of this paper, the medium window size W = 2 is the optimal. An excessively small window leads to insufficient semantic information. An excessively large window introduces redundant information, dilutes the core semantic features, and reduces the efficiency of quantum feature extraction.

Noise in quantum computing hardware severely degrades the performance of the QNN-CBOW algorithm. The lower the fidelity of quantum logic gates, the poorer the quality of outputs generated by QNN-CBOW. For the QNN-CBOW to become practically applicable in the future, the development of high-fidelity, low-noise quantum computing hardware is essential.

The research in this paper has certain limitations, which are mainly reflected in the following three aspects:

- The experiments were carried out based on a classical simulator for quantum circuits and were not verified on the actual quantum hardware. The performance of the model on the actual quantum hardware still needs further verification.
- The circuit design for the quantum layer is relatively simple. This paper adopts a fixed combination of quantum gates and a serial structure without designing an adaptive quantum Ansatz circuit. Therefore, the exploration of quantum feature extraction capabilities is still insufficient.
- The experiments were not verified for generalization on professional-domain long-text datasets (e.g., scientific papers, financial reports). The adaptability of the optimal values of each factor across different domain datasets still needs further research.

While this study provides a systematic analysis of the QNN-CBOW model on a classical simulator, several avenues for future research remain to be explored to advance the practical application of quantum-enhanced NLP:

- Experimental Validation on Noisy Intermediate-Scale Quantum (NISQ) Hardware: Currently, our experiments were conducted on classical simulators that do not account for hardware-specific noise. In the future, we plan to deploy the QNN-CBOW model on actual quantum hardware (such as superconducting or trapped-ion quantum processors). This will involve adapting the quantum circuit architecture to fit the limited qubit connectivity and coherence times of NISQ devices, and evaluating the model’s robustness against quantum gate errors and readout noise.
- Development of Hybrid Quantum-Classical Optimization Strategies: To mitigate the impact of quantum noise and barren plateaus during training, we will investigate advanced hybrid optimization strategies. This includes integrating classical optimization techniques (e.g., adaptive learning rates, momentum-based gradient descent) with quantum-aware algorithms (e.g., Simultaneous Perturbation Stochastic Approximation—SPSA) to enhance the convergence stability and training efficiency of the model.
- Expansion to Large-Scale and Domain-Specific Corpora: Future work will focus on scaling up the model to handle larger vocabularies and longer texts (e.g., scientific papers, financial reports). We aim to explore transfer learning techniques in the quantum domain, where models pre-trained on general corpora can be fine-tuned for specific professional domains to improve generalization performance.
- Exploration of Adaptive Quantum Ansatz: Instead of the fixed serial structure used in this paper, we intend to research adaptive quantum Ansatz circuits (e.g., using different gate combinations or entanglement topologies) to further enhance the model’s capacity to capture complex linguistic features without significantly increasing the circuit depth.

Quantum computing introduces new ideas for the development of natural language processing, and the fusion of quantum neural networks and traditional NLP models represents an important research direction in the future. The research in this paper provides a preliminary exploration of this direction. These findings provide a solid theoretical and practical foundation for the architectural design and parameter optimization of quantum neural networks, thereby advancing the development of quantum natural language processing technologies.

## Data Availability

Data is contained within the article.
